# Minoritarian Labour Welfare in India: The Case of the Employees’ State Insurance Act of 1948

**DOI:** 10.1007/978-3-030-54959-6_5

**Published:** 2020-07-27

**Authors:** Ravi Ahuja

**Affiliations:** Department of Sociology, Bielefeld University, Bielefeld, Germany; Georg-August-University Göttingen, Göttingen, Germany

## Abstract

Through a case study of the Employees’ State Insurance Act of 1948, this chapter examines the historical evolution of a type of welfare schemes in India that made entitlements conditional on specific forms of employment. Global trends in social policy had influenced debates on a social insurance for Indian workers since the 1920s. Transformations of Indian industry, World War II, the post-war crisis and postcolonial economic planning then created conditions for legislation. Just when the international welfare discourse, Indian contributions included, converged on social welfare as a universal citizen right, the regulatory content of the health insurance scheme devised for India diverged from this normative consensus: “Employees’ State Insurance” remained strictly employment-based but also generated horizons of expectation that continue to inform labour struggles.

## Welfare in India: Institutional Pillars and Social Contexts

Welfarism in postcolonial India, in its policies and institutional forms, has not been based on the universal human right to social security that was proclaimed in the centres of metropolitan capitalism in the period following World War II (Nullmeier and Kaufmann 2010; Pierson and Leimgruber 2010). In terms of enforceability, “welfare” never became an integral attribute of citizenship in postcolonial India (Goyal 2013). This blatant historical fact has facilitated the almost complete exclusion of India from the existing scholarship on global welfarism, which, until recently, has been largely confined to the North Atlantic rim.^1^ This exclusion has appeared to be justified even to scholars of South Asian societies who often believe that international policy debates on welfare had largely bypassed India before the end of colonial rule and whose analyses of Indian social policy tend to begin with India’s political independence in 1947 (Goyal 2013). However, key characteristics and global connections of India’s postcolonial social policy become perceivable only if we turn to their—generally miserable—origins in the colonial period, under political conditions of a barely veiled despotism where the State was less exposed to democratic pressures and in less immediate need of legitimizing authority than most European polities.

The colonial Government of India survived, after all, with limited political damage, a chain of famines of genocidal proportions that stretched over the last three decades of the nineteenth century^2^—the very decades when foundations for the European “ welfare state” were laid in response to growing labour movements (Kuhnle and Sander 2010). As late as in 1943, the lack of formal entitlements, or citizen rights, to social protection let the political cost appear bearable to the British authorities of millions of starvation deaths in Eastern India^3^—at a time when the introduction of a system of universal social protection seemed unavoidable on the British Isles even to conservatives. Nevertheless, the last seven decades of colonial rule, beginning with the Famine Codes of the 1880s, were arguably also the period when the foundations were laid for the pillars of Indian social policy as we know it, even though the edifice was fully erected and distinguishable in its present-day form only after the attainment of political independence in 1947. Three such pillars of social policy are distinguishable, which have borne the weight of the—altogether limited—Indian welfarism unevenly, the proportions shifting over time.

The first of these pillars originated from older conceptions of *poor relief*. After the famine crises of the last third of the nineteenth century, these forms of social policy took the shape of targeted “workfare” programmes and, if politically unavoidable, of price controls on essential goods or of provisioning schemes (Brennan 1984). India’s postcolonial and perpetually contentious food rationing systems, the more recent (if now increasingly hollowed out) “National Rural Employment Guarantee” or the “Midday Meal Schemes” for school children are important instances for this policy lineage (Mooji 1998; Amrith 2008; Siegel 2018). A second pillar consists in the establishment of *quotas* regulating the access of specific social groups to public employment and public goods (crucially, education). This pillar, too, originated in the colonial period but assumed growing importance after the end of British rule and particularly when movements of *Dalit* and “other backward” castes became more assertive in the 1980s (Assayag 2012: 451–455; Jaffrelot 2012: 470–476; Srivastava 2018).^4^ This chapter is solely concerned with the third pillar, which made social welfare benefits *conditional on specific forms of employment*. Such policies followed the welfare logic established by the Bismarckian social insurance reforms of the 1880s in that they conceived of welfare entitlements not as a universal right inherent in citizen status, but as derived from legally defined types of employment status and thus as a special right (or, legal privilege) conferred on certain categories of employees.

If the historiography of Indian social policy is meagre in general, it is almost non-existent in regard to employment-based welfare schemes.^5^ To many they would appear, in any case, as being of little consequence to the vast majority of India’s wage-earning population. For 93 per cent of the Indian workforce are conventionally (and somewhat simplistically) reckoned to be employed in the so-called informal sector and thus largely exempted from the ambit of labour law and employment-based welfarism. Even of the remaining 7 per cent about half are reported to be employed “informally” *within the “formal sector”* (Sanyal and Bhattacharyya 2009: 39). They are, in other words, employed by contractors or in other ways that permit to pay much lower wages, evade labour law and withhold employment benefits. Many critical scholars agree with apologists of neoliberalism that India’s labour laws—including employment-based social security schemes—have been relevant only to a small proportion of the country’s workforce and have hermetically sealed off a privileged labour aristocracy of formally employed workers from the vast informal labour economy.^6^

This seemingly uncontroversial line of argument needs to be interrogated, however. At issue is not whether massive social differences exist among India’s workforce: the shrinking proportion of effectively tenured workers in public sector enterprises has undoubtedly very little in common, for instance, with the day labourers constituting the majority of India’s enormous construction labour force. Rather the question is how to conceptualize this wide scope for differentiation among India’s wage-earning people in terms of income, employment conditions, social status and economic security. Like Jan Breman (2013), I would plead for a dynamic, non-dualistic understanding of the phenomenon.^7^ For harsh exclusionary practices and merciless competition between segments of the workforce operate within a structure of *graded informality*: rather than assuming a stationary dichotomy between formal and informal labour status, I propose to examine formalization and informalization *as processes* that are contingent, continuous and contentious. Boundaries and passages between the various segments of the workforce are, in other words, multiple and shifting; they are produced and reproduced through social conflicts and coalitions; and they possess *relative stability* only.

For labour and employment-based social security laws have played a major role not only in the definition of boundaries but also of *passages* between sharply differentiated segments of the workforce. This is not to play down the potency of structural boundaries, which has been reflected in all-too-real difficulties faced by trade unions in developing workable strategies encompassing all sections of the working classes based on a commonality of interest. But taking account also of the passages permits to perceive counter-tendencies, potentials for cross-sectional alliances and to make sense of the persistent demands of informally employed workers to be included in schemes like the Employees’ State Insurance or the Provident Fund. If employment-based social security programmes thus have mattered not only to the fraction of the working classes covered by them explicitly but to wider sections of India’s workforce, it is because they span, together with other labour laws, a horizon of expectation, define possibilities and help to formulate demands.

If we thus assume “(in)formalization” to be a dynamic, bidirectional, even reversible *process*, and that “formality” and “informality” are, accordingly, not to be understood as stable attributes of static and hermetically sealed “sectors” of the labour market, we need to reconstruct this process *historically*. We need to trace, in other words, the historical evolution of those patterns of segmentation within the workforce that came to be described from the 1970s onwards in many parts of the world with the adjectives “formal” and “informal”. Law has served, as Prabhu Mohapatra’s studies show, as a crucial regulatory technology for the separation of “formal” from “informal” modes of employment (Mohapatra 2005, 2012). A historical—as against a merely logical—reconstruction of this process of separation, a chronology of the intertwined processes of formalization and informalization is still lacking, however. As we begin to retrace this chronology, the middle of the twentieth century emerges as a key moment of these processes: almost all major pieces of legislation that have marked out the parameters of India’s postcolonial regime of labour regulation up to the present day were passed during the six years from 1946 to 1952. These acts have regulated labour relations, industrial disputes procedures, trade union rights and also employment-based social benefits, including the Employees’ State Insurance and Provident Fund schemes mentioned already.^8^

Largely ignored by historians, this spate of legislation was, in the immediate political context of the post-war situation, a response of the outgoing colonial administration and their nationalist successors to the extreme political volatility encompassing India in general and to alarming levels of working-class unrest in particular (see Ahuja 2019b). But these changes also had a longer history, dating back to World War I at least, and were conditioned by larger and deeper international as well as India-level contexts: these are discussed more fully in the long version of this essay^9^ and can here only be briefly alluded to. International debates on labour welfare did by no means bypass late colonial India: they provided the “language of welfare” that was used in political controversy and policymaking. Welfarist arguments were taken up both from the debates around the International Labour Organization (ILO) of which India was a founding member and from social policy developments in post–World War I Britain and other Euro-American nation states. Established formats of welfare legislation such as maternity benefit, workmen’s compensation, sickness or old-age insurance were freely borrowed from these international contexts and from the memoranda of ILO councillors. In the same vein, British social policy experts such as John Henry Whitley or William Beveridge came to be involved in legislative processes in India.

While the *discursive and regulatory forms* of early welfarism in India were thus strongly shaped by these international contexts, the specific *regulatory content* (and particularly the strong exclusionary, minoritarian focus) of the emergent regime of labour welfarism was largely determined by India-level contestations between (a) the late colonial and, subsequently, the early postcolonial State, (b) British expatriate as well as Indian big business and (c) an expanding and politically plural labour movement. To cut a long story short, the State became increasingly involved in issues of “labour efficiency” and, therefore, of the social reproduction of the workforce during the World Wars both as a growing industrial employer and as a consumer of strategic commodities; Indian industrialists, on their part, faced increasing international competition and temporary labour market bottlenecks, expanded into more capital-intensive sectors, all of which implied that influential sections of big business came to promote the “rationalization” of industrial labour processes and to acknowledge the importance of raising the living standards of at least sections of the workforce to reduce labour turnover and “absenteeism”; Indian labour movements not only proliferated massively since World War I and came to involve, especially since the 1940s, sections of the workforce even beyond large-scale industry, but they were also a field of contestation between various political forces with a marked presence of militant communist and socialist tendencies.

The historians’ lack of interest in the origins of employment-based social security legislation thus points us towards a wider gap in the historiography of contemporary South Asian societies. The present essay approaches this gap from a specific and limited angle: it traces the prehistory and the making of one major piece of protective labour legislation, the Employees’ State Insurance Act of 1948. This was a compulsory insurance scheme financed by contributions from employers, employees and the state, which was to provide workers employed in “permanent factories” with monetary benefits as well as medical services to protect them from the risks of sickness, childbirth and employment injury while regulating sickness leave also. In this essay, I confine myself to discussing the political and legislative process from which this piece of labour legislation emerged and how it both gave legal expression and contributed to an increasing differentiation among the industrial workforce along the lines that would later be described in terms of a “formal”-“informal” divide.

The chapter is organized as follows. The section “Early Industrial Welfare and the Debate on Welfare Legislation in Interwar India” traces early industrial welfare schemes at the company level and discusses why the reproduction of the industrial workforce emerged as a political issue at the all-India level during the interwar period. The following section “The Employees’ State Insurance Act: The Making of a Law” traces the making of the Employees’ State Insurance Act in the political field of forces of the transitional 1940s. The concluding section “Repercussions: Graded Informality, a ‘Birthright’ Lost and a Horizon of Expectation” argues that the results of this process were contradictory: while the specific form of Indian health insurance contributed to a harsh segmentation of the working classes, and while the promise of welfare as a citizen’s right remained unfulfilled, horizons of expectation were spanned simultaneously that continue to inform struggles for social equality.

## Early Industrial Welfare and the Debate on Welfare Legislation in Interwar India

Before World War I, employers both British and Indian, colonial officials and large sections of the press agreed that India did not require protective labour legislation or a welfarism focused on industrial labour. Two lines of argument stood out, the first asserting that India’s young industry could not afford expensive welfare measures if it was to compete internationally. A leading nationalist newspaper, *Amrita Bazar Patrika*, formulated this position with admirable clarity in 1875: “A larger death rate amongst our operatives is far more preferable to the collapse of this rising industry. […] We can, after the manufactures are fully established, seek to protect the operatives”.^10^ The second line of argument insisted that labour welfare was largely irrelevant to India since the country had “as yet practically no factory population, such as exists in European countries” (Indian Factory Labour Commission 1908: 18). Factory work, the adherents of this latter view reasoned, was no more than a temporary occupation of and a supplementary source of income for a migratory workforce. The mill worker, it was held, was “essentially an agriculturalist”: “His heart is in the country and not in his work” (Burnett-Hurst 1925: 60). Moreover, the workers’ health was provided for by their rural families and other “traditional” village-based forms of mutual aid: “in most cases” the Indian Factory Labour Commission asserted in 1908, “he is secured against want by the joint family system” (p. 19) and an official report on the industrial city of Bombay concurred in 1923 that periodical visits to the home village had “a beneficial effect upon their health as reflected by weight and counteracts to a very large extent the effects of working and living conditions”.^11^

Such opinions continued to be pronounced well into the postcolonial period, and cracks appeared, at first, in this hegemonic construct only in certain industrial sectors and for limited periods. Exceptions were capital-intensive industrial enterprises such as the Tata steel works, founded in 1907 and operational by 1912, where profits depended on the stable employment of a skilled workforce and where—as in the case of the railways and their extensive engineering workshops—strategic needs of the empire were at stake. Here welfare schemes, including housing and health services, were created even before World War I and expanded in the course of the 1920s (Lala 2006: 284; RCLI 1931a: 53–69 and passim). Other exceptions were observable even in India’s more typical labour-intensive industries when severe bottlenecks of labour supply threatened to stifle industrial production for periods sufficiently long to affect capital returns. Bombay’s cotton textile industry experienced such a bottleneck during the plague crisis of the late 1890s. This induced industrialists to look for devices restricting the mobility of the workforce—devices that included company-level welfare measures and, more particularly, housing programmes (cf. Sarkar 2018: 202–211). When the exigencies of the “Great War” dictated a greater British reliance on India’s labour markets and industries, the cracks widened and the “welfare” of industrial labour emerged, for the first time, *as a political issue at the all-India level*. The Indian Industrial Commission (1916–1918) was appointed by the Government of India with no labour representative, while half of its members were Indian or British businessmen. Interestingly, the Commission moved cautiously away from the earlier consensus in its report: the lack of welfare facilities that addressed dismal health conditions among the industrial workforce was now identified as a competitive handicap since even the cheap wages of Indian workers could not make up, the Commission believed, for their alleged low “efficiency”:

The conditions under which industrial operatives live and work in this country ought, if efficiency be aimed at, to approximate, as nearly as circumstances permit, to those of temperate climates. […] The problem, not only on moral grounds, but also for economic reasons, must be solved with the least avoidable delay, if the existing and future industries of India are to hold their own against the ever-growing competition, which will be still fiercer after the war. No industrial edifice can be permanent, which is built on such unsound foundations as those afforded by Indian labour under its present conditions. (Indian Industrial Commission 1918: 179f)

The recommendation of measures implying legal obligations on the part of employers was, however, carefully avoided. Even so, the argument of welfare as a precondition for “efficiency” stuck and was taken up time and again in the following years. Accordingly, in April 1922, a report published in *The Servant of India*, mouthpiece of an influential social reform society, summed up the rationale of India’s first “Industrial Welfare Conference” as follows:

Welfare work wherever conducted on right lines has been found to be a veritable boon to the worker and a sound business proposition to the employer. India cannot hope to compete successfully with other countries unless the present low level of efficiency of the Indian labour is considerably raised. (Kanekar 1922: 136)

Unprecedented levels of labour unrest and the emergence of numerous trade unions after the “Great War” added the desideratum of “social harmony” to that of “efficiency”. This induced Gandhi, for instance, to offer a nativist justification for welfarism by developing during these years the idea of a paternalist “trusteeship” that employers were morally obliged to take upon themselves for the benefit of their employees (Chandavarkar 1998). During the interwar years, a growing minority of industrial employers came up with voluntary factory-level welfare schemes that addressed issues of social reproduction by providing for housing, crèches, educational facilities, subsidized grain shops, credit or dispensaries. These generally modest schemes often had a sharp disciplinary edge, as they sought to suppress the militancy and to reduce the horizontal mobility of core segments of the factory workforce. They were particularly prevalent in areas with large concentrations of industrial employment such as the cotton textile metropolises of Bombay and Ahmedabad, where strikes and unionization were on the rise and where sizeable local labour markets enabled workers, at least in boom years, to shift to employers who offered better conditions (RCLI 1931b: 260f).^12^

A “social policy” in the sense of a legal regulation of the workforce’s social reproduction based, in part, on statutory contributions by employers was resisted, however, on the whole successfully throughout the interwar period. As in other countries (Kuhnle and Sander 2010: 71–74) a “Workmen’s Compensation Act” was the first piece of labour welfare legislation in colonial India. It was enacted in 1923 and put into force a year later. The divergence between European and Indian legislation was already perceivable, however, as the scope of workmen’s compensation was defined much more narrowly than, for instance, in Britain. Small-scale industries and agriculture (including the sizeable quasi-industrial plantation complex) were expressly exempted from the law (Punekar 1950: 55). Numerous loopholes were created even with regard to labour market segments covered by it, which included initially not more than four million workers (ILO 1957: 95). Moreover, the implementation was left to district magistrates in general; even 20 years later, a special commissioner to adjudicate workmen’s compensation cases had been appointed solely in Bombay, while in Bihar, Bengal and Madras, Labour Commissioners were charged with workmen’s compensation as an additional duty (Punekar 1950: 73). As a result, the “Compensation Courts” appear to have sat in some of India’s vast Provinces only twice a year (Health Survey and Development Committee 1946: 77). Even more importantly, adjudication could only begin after private negotiations between the injured worker and the employer had failed, which made it almost impossible for uncounselled workers to stake their compensation claims. The injured worker, it was observed, ran “the risk of losing his job on top of losing a limb, if he decides to fight a case” (Adarkar 1947: 14). Even when adjudication took place, after all, the actual payment of the awarded compensation was often not enforced (Lokanathan 1929: 107; Punekar 1950: 74).

When the economics professor and government councillor B.P. Adarkar submitted his “Report on Health Insurance for Industrial Workers” to the outgoing colonial dispensation in 1945, he recommended to scrap the Workmen’s Compensation Act altogether. It was to be replaced by an integrated healthcare scheme, as the working of the former had been “far from satisfactory”. He had very similar remarks for the other major item of interwar labour welfare legislation, that is, the Maternity Benefit Acts that had been passed in the majority of provinces after Bombay made a start in 1929 (Adarkar 1945: 10; s.a.: ILO 1957: 100f). The Report of the (Rege) Labour Investigation Committee similarly observed in 1946 that the

main defects of maternity benefits legislation are that it is neither uniform nor universal, that there is no provision for free medical aid before, during or after confinement except in a few provinces […], and that there is no provision for preventing an employer from dismissing a woman worker on the first sign of pregnancy except in a few provinces.

The Report also stated that women workers had often been dismissed when the Acts were put in force and that employers continued to “show a preference for the employment of unmarried girls, widows and women past child-bearing age” (Labour Investigation Committee 1946: 57). In a circular letter to the provincial governments in May 1945, H.C. Prior, Secretary to the Government of India, concurred that the Workmen’s Compensation and the Maternity Benefit Acts had “serious defects […] which cannot be removed except by means of an integral scheme of insurance”.^13^

Even though employers resisted any legal obligation to pay for welfare measures as an interference with the freedom of labour contract, the demand for social security legislation became if anything more insistent from the late 1920s onwards. The report of the Royal Commission on Labour in India (RCLI), presented to the British Parliament in 1931, accepted the view that continuing circulatory migration between city and countryside distinguished Indian factory workers from their European counterparts, but refuted the conventional notion that the former were “essentially” peasants (RCLI 1931b: 11–13). They conceded that the “villages have hitherto provided a measure of insurance against the effects of the various changes which may reduce, interrupt or destroy the earning capacity of the worker” (p. 19). Yet, they added, this “measure of insurance” did not prevent workers even after short periods of illness from falling into debt and from finding themselves “destitute of resources, unable to take proper measures to restore [their] health and in difficulties regarding even the means of subsistence” (p. 265). The RCLI thus recommended the development, in due course, of a system of sickness insurance for industrial workers, funded by contributions from employers as well as employees (pp. 265–269). However, one of its members, N.M. Joshi, a prominent social reformer, legislator and trade union leader with close links to the ILO in Geneva, went beyond this Bismarck-style employment-based conception of social security: in February 1932, the consultative Committee of the Round Table Conference discussed (and promptly turned down with reference to “the peculiar conditions of India”) his suggestion

that the chapter on Fundamental Rights in the new Reformed Constitution for India should include a clause entitling every citizen to support from public funds, if no work could be found for him and to the provision, through a system of State insurance or otherwise, for maintenance during sickness, infirmity or old age and in the case of women for a reasonable period before and after confinement.^14^

The oppositional Indian National Congress, too, endorsed demands for social welfare: during the campaign for the provincial elections in 1936–1937, its manifesto promised “protection against the economic consequence of old-age, sickness and unemployment”.^15^ A National Planning Committee (NPC), instituted by the National Congress in the late 1930s, was chaired by the moderate socialist Jawaharlal Nehru, but it also included a strong posse of Indian businessmen. In 1940, the NPC resolved that “ social security should be assured to all classes of workers” (National Planning Committee 1940: 60). More specifically, the NPC announced that “[a] system of compulsory and contributory social insurance for industrial workers should be established directly under the control of the State, to cover the risks of sickness and invalidity”. This as well as other schemes were envisaged for independent India and to “be extended by stages, priority being given to particular classes of workers, with due regard to the relative urgency of their needs, facility of application, and to the ability of the community to provide for them” (p. 55). The NPC thus applied the globally circulating term “ social security” to the Indian context as early as in 1940^16^ but limited it, in the main, to labour welfare: it made entitlements to social benefits conditional, at least initially, on industrial employment and did not seek to establish them as a constitutional citizen’s right. It thus chose to follow the RCLI’s more restricted Bismarck-style approach rather than N.M. Joshi’s universalism.

With the Indian National Congress assuming in 1937 the government of the majority of provinces, including Bombay with its sizeable industrial centres, the latter emerged as the main hub for the development of a nationalist labour policy. In their “Labour Programme” of August 1937, the Bombay government already dampened the expectations raised during the election campaign^17^ and had their Labour Commissioner declare a few months later that “conditions do not at present exist in the Presidency for the successful operation of a scheme of sickness insurance as it is understood and worked in the United Kingdom or other foreign countries”. As preliminary, cautious steps, the government proposed to first generate the required statistics, to legally sanction three to four weeks of paid sick leave for industrial workers and to deduct part of the wages for this leave period in order to create a State-administered provident fund. These were seen as first steps towards a social insurance scheme that was to be restricted to industrial workers.^18^

## The Employees’ State Insurance Act: The Making of a Law

When the colonial government declared India’s war entry in September 1939 without consulting the nationalist opposition, the provincial governments led by the Indian National Congress stepped down. In 1942, Nehru and other nationalist leaders were arrested, and the National Planning Committee ceased to function. The Congress’ temporary exclusion from policy-making circles did not, however, cut short the debate on social insurance for industrial workers. Discussions between employers’ spokesmen, trade unions and representatives of the Government of India on a compulsory sickness insurance continued during the early years of the war, and if they remained fruitless, this was justified now more often by a lack of funds rather than by asserting the undesirability or the irrelevance to India of such a scheme.^19^

This political impasse ended only in the latter half of 1942, when the colonial suppression of the nationalist Quit India Movement was in full swing and when the British War Cabinet discussed the expediency, under these circumstances, of a “more progressive social and industrial policy” in India.^20^ The Government of India’s new Labour Member B.R. Ambedkar, a towering leader of India’s “untouchables”, used this situation to suddenly announce a social insurance bill that would be introduced in the Legislative Assembly by spring 1943.^21^ B.P. Adarkar was appointed by the Labour Ministry to develop a “Report on Health Insurance for Industrial Workers”, which was submitted in the fall of 1944.^22^ The Report drew upon various tentative schemes developed in the preceding years (Adarkar 1945: 14–16) but developed a concrete financial and administrative structure for the first time. It was vetted subsequently by ILO experts at the request of the Government of India and revised by Adarkar by July 1945.^23^ The Workmen’s State Insurance Bill was introduced in the Central Legislative Assembly towards the end of the following year and eventually passed as the Employees’ State Insurance Act (ESI Act) in March 1948. It was to provide workers in perennial factories with more than ten employees with eight weeks of paid sick leave, monetary benefits in case of maternity, accident and invalidity, as well as medical benefits to be offered by special medical services that were to be created for the purpose.^24^

The law remained a dead letter for several years, however. The *Times of India* observed a year after the passing of the Act that while the ESI depended on the close cooperation of the Central Government, the Provincial Governments and of employers, “[a]ll the three are too preoccupied with their own problems to attend to the teething troubles of a child whom none consider as their own”.^25^ In 1951, the All-India Organisation of Industrial Employers, a body representing Indian big business, demanded a further postponement of its implementation and particularly of any raising of contributions from the employers since they considered the scheme “socially unjust” and even “disastrous” in its economic consequences, as it imposed “on industry a burden which it cannot bear”.^26^ Influential forces within the ruling Indian National Congress supported this stance: the government of the populous northern province of Uttar Pradesh had previously “signified their opposition to the scheme following a representation made to the Centre Government by certain employers at Kanpur”—the *Times of India’s* commentator even feared that the project was about to be abandoned.^27^ In 1952, Jawaharlal Nehru finally inaugurated a pilot scheme that was confined, however, to Delhi and the industrial city of Kanpur.^28^ It was only in the course of the late 1950s that ESI coverage was rolled out more widely though considerable parts of the country still remained outside the remit of the Act. A full decade after the ESI Act had been passed by the Parliament of independent India, a “Study Group on Social Security” set up by the Ministry of Labour believed that of 2.2 million factory workers to which the law extended, a mere 1.3 million were actually covered by the mechanisms of implementation established by then (Study Group on Social Security 1958: 25).

The passing of the law had clearly not put a stop to the struggle over health insurance for workers—trench warfare over legislation now merely turned into an unending battle of attrition over its implementation. While these struggles over implementation require further research, we can here only discuss schematically four major lines of contestation that emerged already in the process of legislation in negotiations between business representatives, trade unionists and state officials. These were (a) the compulsory nature of the scheme, (b) its contributory character and the connected issue of financial liability, (c) its administrative structure as a *state* insurance and (d) the vexed question of scope.

Backed up by the 1944 Recommendation of the ILO, government officials and experts as well as trade union spokesmen agreed that sickness insurance for industrial workers had to be *compulsory* if it was to have any impact (Punekar 1950: 3,9; Adarkar 1945: 24).^29^ This caused some discomfort in business circles and even among industrialists who admitted a certain need for an improvement of health services for industrial workers. At issue was not only that compulsory participation in a health insurance scheme implied an obligation to contribute to it financially—an aspect we shall discuss instantly. Employers were also concerned that a compulsory scheme regulating, among other things, the employees’ right to paid sickness leave brought the conditions of work or the modalities of the performance of the labour contract under the scrutiny of state officials. Employer spokesmen thus pointed out repeatedly that the issue of health insurance was directly connected to that of holidays with pay^30^—the scheme was thus not external to the labour relationship, but a mechanism that restricted the “freedom” of employers to fashion labour contracts as they pleased. In 1935, the Bengal Chamber of Commerce had flatly opposed any social insurance scheme based on compulsion, had suggested voluntary schemes at the company level according to a “model scheme” to be drawn up by government and had demanded that existing “adequate” arrangements should not be interfered with.^31^ By 1943, when many employers recognized that a compulsory sickness insurance scheme could be forestalled for some time but not avoided altogether, the Bombay Chamber of Commerce still demanded that existing voluntary employer schemes should be allowed to continue, thus opening a route towards the exemption from legal obligation.^32^ Trade unionists insisted, on their part, that “[e]xceptions in favour of any private factories will lead to unfair practices”.^33^ Adarkar, while recommending strictly regulated exceptions wherever employers had created satisfactory insurance schemes for their workforces, emphasized that such schemes were extremely rare: “The existing medical facilities in most places are no doubt extremely inadequate; even some of the so-called health insurance schemes are a mere parody of what they should be” (Adarkar 1945: 186). At the end of the day, employers could not prevent that the ESI Act defined health insurance as a compulsory scheme. The interdependence between employment-based health insurance and the conditions of the performance of the labour contract was even brought forward openly by B.R. Ambedkar as an argument for the urgency of regulating employment conditions through the Standing Orders Act that was passed by the Central Legislative Assembly in 1946.^34^

The second line of contestation emerged over the *contributory character* of the ESI scheme and the connected issue of *financial liability*. From the early war years onwards, state officials and trade union representatives had agreed that the envisaged social insurance scheme needed to be based on contributions by employers and employees. Business spokesmen and trade unionists had, at the same time, concurred in the opinion that a financial contribution by the State was required.^35^ Raising the threshold for the passing of an unwelcome law without having to contradict government openly was surely one of the tactical considerations that prompted employers to pursue this line. The War Government at the central level responded by taking the comfortable stand that if state subsidies were required, they would have to come from the provinces—predictably, the latter ruled this out altogether (Adarkar 1945: 164).^36^ The Adarkar Report, in 1945, argued strongly for a financial contribution by the State, but it outlined two alternative models of funding, only one of which involved state subsidies (Adarkar 1945: 38–45, 105–109). The Report also advocated a state guarantee for the solvency of the social insurance scheme (Adarkar 1945: 63f)—a demand taken up by the Bombay Millowners’ Association when the Bill was eventually under discussion in the Constituent Assembly, in order to protect the employers from financial liabilities.^37^ As per the Act finally passed a year later, the Government of India undertook to cover two-thirds of the administration costs for the first five years, while the provinces were asked to finance one-third of the costs of the medical facilities that were to be established for the provision of the medical benefits. An estimate calculated that, on this basis, employers were to contribute 60 per cent of the total ESI budget, while employees and the State were answerable for 20 per cent each (Punekar 1950: 194f). Again, the actual implementation of the Act created a rather different scenario: business advocates achieved a temporary exemption of companies from the payment of “maximum contributions”,^38^ and by the end of the 1950s, trade unions calculated that employees had, in fact, contributed significantly more to ESI funds than employers.^39^ The state governments, on their part, renegotiated their share of expenses for the ESI scheme’s medical services and succeeded in reducing it from one-third to one-quarter (Albuquerque 1958: 108f).

Since state contributions remained narrowly circumscribed, the operational costs of the ESI scheme were mainly borne by bipartite contributions from employers and employees. The *administrative structure*—the third line of contestation we need to take account of—assumed a strongly tripartite form, however, and came to be dominated by state officials: while employers’ associations and trade unions were entitled to appoint their representatives, the administrative bodies in control of the ESI funds were controlled by government servants as were the special arbitration structures for ESI disputes (Albuquerque 1958: 108; Punekar 1950: 148–150, 158f). This was a major departure from the implementation structure of the Workmen’s Compensation Act where the respective employer was in charge of payments to beneficiaries and where hurdles had been created intentionally, as we have seen earlier, to render recourse of claimants to legal adjudication more difficult. The state-centred administrative structure of the ESI was created with the explicit aim of preventing the malfunctions of the earlier Acts that were believed to be rooted in the principle of “employer liability”: “for if the employer is saddled with the responsibility of compensation, he is bound to find ways of avoiding it” Adarkar 1947: 14, see also 11–17, 23, 1945: 12).

The fourth and even more defining line of contestation arose in regard to the issue of *scope*. When the debate grew more intensive in 1940, an alliance of British and Indian industrialists demanded that a compulsory social insurance scheme, if it had to be created at all, was to have an extensive reach from the start. They insisted to include the Princely States—comprising almost one third of the subcontinent—to avoid unfair competitive advantages for industrialists operating from these territories.^40^ For the Bombay Chamber of Commerce, this was an issue sufficiently important to justify the “postponement of the scheme for several years”.^41^ Sir Vithal Chandavarkar, spokesman of the Employers’ Federation of India, even combined his appeal to the new government of independent India not to “scare away private enterprise” with the demand that the undue focus of labour legislation on industrial workers should be overcome and that its scope needed to be extended to agricultural workers. Ostensibly in the best interest of the working classes, such proposals seemed to have the main objective of derailing the project by raising the hurdles.^42^ Adarkar envisaged a universal scheme in the long run, but recommended for the initial period a narrow focus on workers in perennial (i.e. non-seasonal) factories in three industrial sectors that had employed about 1.3 million workers in 1942: textiles, engineering, “minerals and metal” (i.e. the metallurgical and oil industries) (Adarkar 1945: 29, 159). This would have covered about 60 per cent of the factory workforce.^43^ The All-India Trade Union Congress (AITUC) demanded a wider scope to include all employees of “organized industries”, irrespective of occupation, whether working in factories or not, and including employees of seasonal factories (e.g. those processing agricultural produce like sugar) as well as “[s]ome of the dependents”.^44^ Two ILO experts, Raghunath Rao and Maurice Stack, were assigned the task to revise the scheme and recommended to extend it to all factory workers in perennial factories.^45^ This recommendation was incorporated into Adarkar’s final report and subsequently in the Employees’ State Insurance Act of 1948.^46^

Consequently, smaller manufacturing units, agricultural labour, including the workforce of India’s sizeable quasi-industrial plantation economies, the enormous construction sector as well as miners and transport workers remained outside the remit of the ESI Act, though it allowed provincial governments to expand its scope. Nor were the workers’ families initially covered by the health insurance. Furthermore, provincial governments were empowered to grant exemptions from the law to industries considered to be unable to contribute to the scheme. The provisions for the Act’s implementation allowed for further exceptions even within the industries explicitly covered by the Act. Crucially, “the conditions of qualifying period for cash benefit exclude[d] casual workers”, while unpaid apprentices were not granted protection because the Act applied to remunerated labour only (Punekar 1950: 84).

## Repercussions: Graded Informality, a “Birthright” Lost and a Horizon of Expectation

While the ESI Act thus permitted a differentiation of employment conditions even on the same shop floor, it also contributed to a process of differential formalization that generated a pattern of graded entitlements in the labour market and multiple rifts among the working classes as a whole. Certain sections of the workforce had, for instance, access to health facilities that were and would remain far superior to the ones provided by the ESI scheme—this was the case with the nationalised railways, which had generated their own medical services in the course of the 1920s, and it would hold true for new public sector enterprises of Nehruvian India.^47^ Other and much larger sections of the industrial workforce were, at the same time, legally entitled only to a level of sickness and invalidity protection much inferior to that offered by the ESI. This becomes evident if we return to the Workmen’s Compensation Act of 1923, which the ESI Act was to replace. In fact, it did so only for about a third of the six million workers that were entitled to workmen’s compensation by the early 1950s. The incongruent remits of the two laws thus implied that the industrial workforce of postcolonial India was further divided into a minority segment entitled (by the ESI Act) to *pensions* in the event of work accidents and another segment, twice as large, entitled (by the Workmen’s Compensation Act) to *lump sum payments*, which provided not only less security but were also more difficult to claim (ILO 1957: 95, 104; see also Punekar 1950: 54–57, 154–158).

This is only one of many similar instances, one element of a much larger phenomenon: multiple and overlapping central and provincial labour laws have defined the “workman”, the “worker” or the “employee” and, accordingly, their remit in sharply diverging ways, while labour tribunals and courts of justice have added to the complexity of these definitions by way of conflicting interpretations (Karuna 2019).^48^ The Employees’ Provident Fund Act of 1952 applied, on its part, only to about half of the factory workers covered by the ESI Act of 1948 (Narasimhan 1953: 49). The incongruence of labour- and employment-based welfare laws in terms of scope thus generated a complex site of conflict that was to engage employers, trade unions, judges, government officials and various other social actors for the decades to come: legislation did not result in a formal/informal bifurcation of the workforce into two “sectors”, but in an unstable, contested and to some extent malleable structure of graded (in)formality.

Despite its narrowly confined remit, postcolonial India’s first Labour Minister, Jagjivan Ram, celebrated the ESI Act, when it was passed, as a breakthrough: “the tiny and tender sapling” would “in its own time, grow into a gigantic tree” and the scope of the scheme would be “extended gradually and steadily so that ultimately it becomes all-comprehensive”.^49^ Health Minister Rajkumari Amrit Kaur confirmed that the scheme was to be extended, in due course, to about 85 per cent of the population.^50^ A propaganda film, released by the Government’s Films Division in 1952, pronounced that the ESI scheme would be extended “until its benefits are available in every industrial section of our country, until not only our 2.5 million workers, but all employees, including our agriculturists, enjoy this, their birthright”.^51^ The choice of these words, read out in the King’s English as the footage shifted from machine operators to ploughing farmers, was significant: “swaraj [self-governance] is my birthright” had been a rousing slogan in the independence struggle, associated with the militant nationalist Bal Gangadhar Tilak. Quoting this phrase elevated security from illness to the status of a fundamental right intrinsic to *citizenship* of independent India. The universalization of health insurance from an employment-based privilege to a citizen right was thus announced and explicitly married to the nation-building project. This official promise survived the bleak facts of implementation for quite some time. In 1965, when V.V. Giri’s influential *Labour Problems in Indian Industry* was reprinted once again, the former Labour Minister and future President of India still characterized ESI as “a nucleus of a general social insurance scheme” (Giri 1965[1959]: 267; see also Albuquerque 1958: 108).

However, the universalization of health insurance turned out to be, as we know today, one of the unfulfilled promises of postcolonial citizenship and was postponed ad infinitum. As late as in 2013, a mere 3 per cent of India’s almost half billion–strong workforce or less than half of the “organized sector” workers were entitled to often unsatisfactory ESI benefits (Duggal 2015: 19). Instead, the Employees’ State Insurance Act came to be one of the key mechanisms for the separation of India’s formal and informal labour economies into an embattled, segmented structure of graded entitlements. Despite the many references to Beveridge and Geneva, the guarantee of a national minimum standard did not diffuse to India in subsequent decades as the basis of a universalist welfare policy. Employment-based Indian welfarism remained robustly minoritarian, confined to a very small and mostly male section of those who built postcolonial India for wages. When consulted on the ESI scheme by the outgoing colonial government in 1945, William Beveridge had stated that “freedom from want [was] probably to be sought for the greater part of the Indian population” in a “different direction”. This not only was prescient but also indicates that even liberal British reformers did not rule out, at the time, the *possibility* in India of social policies more far-reaching and comprehensive than those the postcolonial dispensation chose to accept as inheritance from their colonial and deeply conservative predecessors.^52^

Yet the lines, drawn by labour welfare laws of the 1940s and 1950s, should not be understood solely as borders breaking the workforce into a multiplicity of fragments but also as a horizon of expectation—a horizon that would remain out of reach for most workers but has been well in sight: it has created a language for the formulation of standards of “decent work” that are transgressively utopian as well as eminently carnal in their concreteness. They have thus endowed labour struggles with a moral edge and could even serve as blueprints for legislative initiatives for the protection of informally employed workers. A particularly striking case in point is the Dock Workers Act of 1948, which served as a template for mobilizations among the enormous and almost completely “informal” construction workforce for the establishment of welfare boards and for the (no doubt severely diluted) Unorganized Workers’ Social Security Act of 2008 (Dietrich 1992: 1970–1972; Agarwala 2013: 4, 47–49). Similarly, contract workers and employees of predominantly “informal sector” industries have for many years raised the demand for inclusion in “ESI”^53^—a demand that has signified not only the desire for protection against the existential risks and economic perils of sickness. For entering the remit of one of India’s employment-based welfare laws—such as the ESI or the Provident Fund—also implied a formal acknowledgement of their employment status. This was no negligible achievement since certified employment status could serve as a legal basis for claiming further entitlements. Table 5.1. summarizes the analysis.

**Table 5.1:** Social protection in India, 1880s–2014: changing ideas and policies

Period	Changes in politics and society	Social question, social ideas and problem groups	Broader ideational frames and contexts	External ideas and models	Key actors	Policies, legislation and programmes
1880s–1910s	Consolidated colonial rule and emergence of nationalism	Priority: famine question Minor: women’s and labour questions	Free trade imperialism, racism, and Malthusianism	Poor law	Colonial state; early nationalists	Priority: Famine Codes
1920s–1970s	Popular nationalism; social movements; World Wars; Great Depression; Independence; and economic planning	Priorities: labour question (in key sectors) and food security. Minor: poor relief; public health; and caste discrimination	Nationalism; etatism and planning; development; corporatism	Social insurance; tripartism; rationing systems; and citizen right to social security	Late colonial state; nationalism; postcolonial state; Indian big business; labour and other social movements	Priorities: minoritarian labour welfare; rationing system for basic goods; basic healthcare
1980s–2014	Rise of new political formations (end of Congress dominance); “liberalization”; and increasing caste tensions	Priority: caste question; shifting to rural welfare towards the end Minor: declining labour welfare	Neoliberalism; critique of caste; Hindu nativism	Neoliberal “deregulation”; towards the end: recurrence to “new deal” policies	Postcolonial state; regional forces; lower caste and Hindu nativist movements; Indian big business	Priorities: quota systems of social redistribution and workfare programme in rural areas

*Note:* (a) Notions of “ social protection” have a much longer history in India than the one indicated in the “periods” of the Table, which refers merely to the emergence of codified social policies covering large parts of the subcontinent. (b) All periodizations must be provisional, given the unsatisfactory state of historical research, and tentative, given the simultaneity of contradictory tendencies. (c) The governments under Narendra Modi since 2014 appear to usher in a new phase when older welfare structures are dismantled and those supposed to take their place are not yet clearly discernible. The political and economic impact of the Covid 19 crisis seems to trigger a further informalisation of employment patterns thereby exacerbating the decay of employment-based social protection. However, no schematic representation is possible at present

Employment-based social security schemes thus have mattered not only to the minority of the Indian workforce covered by them explicitly but to wider sections of the working classes, as they have defined possibilities, together with other labour laws, and have helped to formulate demands. While undeniably dividing wage earners according to graded entitlements, these schemes have simultaneously established a form for collective claims of workers against employers and a sense that the State bears an—if often shirked—duty to guarantee such claims. This sense of entitlement has made it difficult to do away altogether with the Employees’ State Insurance and other labour welfare schemes despite three decades of largely unmitigated neoliberal policies in India. However, in a significant new development, the second Modi government introduced to the Indian parliament in late 2019 a “Social Security Code”, which aims at “amalgamating” and “corporatizing” eight existing labour welfare schemes, including ESI, while simultaneously reducing compulsory contributions. It remains to be seen whether this new law will, in fact, do more than undermine the existing, doubtlessly minoritarian welfare schemes (mainly, as some trade union argue, by transforming legal entitlements into benefits to be doled out by the executive at their discretion) and achieve the proclaimed goal of expanding health services to larger parts of the workforce.^54^

## Footnotes

1 This is exemplified by paradigmatic studies such as Esping-Andersen (1990). The more recent Oxford Handbook of the Welfare State (Castles et al. 2010) does present a section on “Emerging Welfare States” that contains chapters on Latin America, East Asia, Eastern Europe and Russia, but still not on South Asia.

2 There is now a rich historiography of famines in India that includes the important monographs by Bhatia (1991, 3rd ed) and Davis (2001).

3 This has not been lost on either contemporaries or later scholars. See, for example, Nehru (1989: 496) (1st ed. 1946) and Sen (1981).

4 See also Chap. 10.1007/978-3-030-54959-6_4, in this book.

5 Even the historian of Indian labour perhaps most sensitive to issues of social security assumed that state-born welfare schemes were largely irrelevant in colonial India, Das Gupta (1994: 612–620).The only colonial welfare laws that have attracted more serious historical analysis so far are the provincial Maternity Benefit Acts and, more particularly, that of Bombay (cf. Chhachi 1998; Srivastava 2018).

6 See, for example, Parry (2013: 45), where formal sector workers are characterized as a “complacently cocooned enclave of labour inhabiting a ‘citadel’ of state-sponsored privilege, progressively protected against incursions by workers from outside”.

7 Breman (2013; see esp. Chap. 10.1007/978-3-030-54959-6_1) has summed up and updated his argument, first presented in 1976 in a series of articles.

8 For an overview, see International Labour Organization—ILO (1957).

9 For a detailed discussion, see Ahuja (2019a), especially sections “The Employees’ State Insurance Act: The Making of a Law” and “Repercussions: Graded Informality, a “birthright” Lost and a Horizon of Expectation”.

10 *Amrita Bazar Patrika,* 2 September 1875, quoted in Chandra (1966: 336).

11 T. Maloney, *Report on Humidification in Indian Cotton Mills,* Delhi: Government of India Press, 1923, quoted in Burnett-Hurst (1925: 60, fn. 1).

12 See also: *Bulletins of Indian Industries and Labour 63: “Sickness Insurance”,* Government of India: New Delhi, 1937, p. 82 and Srivastava (2018).

13 H.C. Prior, Secretary to the Government of India, to all Provincial Governments, 4 May 1945 (“Subject: Health Insurance Scheme”), BL: IOR/L/E/8/4948 (“Health Insurance for Industrial Workers, incl. Sickness Statistics”).

14 ILOrep 3/1932, p. 65 (citing *The Hindu*, 1 March 1932). The Roundtable Conferences prepared the Government of India Act of 1935, which was the last quasi-constitutional legal framework of colonial rule before independence.

15 “Industrial Reform in Presidency”, *ToI,* 18 August 1937, p. 6.

16 During the interwar period, the phrase “ social security” had circulated in the Indian press with increasing frequency, especially after the US “Social Security Act” of 1935, but mainly with reference to international developments. From the beginning of the 1940s onwards, the term was applied to Indian contexts more regularly. For evidence, see, for instance, the digital archive of the Times of India. The quoted instance from the resolutions of the National Planning Committee in 1940 might be significant in this respect.

17 “Industrial Reform in Presidency”, *ToI,* 18 August 1937, p. 6.

18 *Bombay Chronicle,* 15 November 1937, p. 8 (“Sickness Leave with Pay for Industrial Labour”) and p. 6 (“An Essential Reform”); see also ILOrep 11/1937, pp. 18f.

19 See, for example, ILOrep 1/1942, pp. 4–6 (special reports produced on the basis of participants’ minutes and papers distributed at the 3rd Labour Ministers’ Conference).

20 BL: IOR/L/E/8/2527 Social Reform in India, 1942–1944. See also above.

21 ILOrep 10/1942, p. 5 (cites *The Statesman,* 14 October 1942).

22 ILOrep 10/1944, pp. 24f (cites “Unofficial Note issued by the Bureau of Public Information, Government of India”, n.d.).

23 ILOrep 7/1945, p. 18 (referring to *Indian Labour Gazette [hereafter: ILG]* 6/1945).

24 *ILG* V/10 (April 1948), pp. 698–701; ILOrep 4/1948, pp. 90–93 (cites *The Statesman,* 2 and 3 April 1948).

25 “Employees’ State Insurance Corporation”, *ToI,* 30 August 1949, p. 11.

26 ILOrep 5/1951, pp. 16–18 (cites *Hindustan Times,* 17 May 1951).

27 “State Insurance Scheme. Employers’ Opposition”, *ToI,* 18 August 1950, p. 5; “A Sorry State”, *ToI,* 20 September 1950, p. 6. See also: Albuquerque (1958: 108f).

28 ILOrep 2/1952, pp. 94f (cites *The Statesman* and *National Herald*, 25 February 1952).

29 See also *Trade Union Record,* IV/5–6 (1–2/1945: 38f).

30 ILOrep 8/1940, pp. 35f (cites Circular no. 146, dated 8 August 1940, of the Employers’ Association of Northern India in Kanpur); ILOrep 6/1943, p. 12 (citing Proceedings of Bombay Chamber of Commerce for May 1943).

31 ILOrep 11/1935, p. 12 (“Abstract of Proceedings of the Committee of the Bengal Chamber of Commerce for Sept. 1935”).

32 ILOrep 6/1943, p. 12 (excerpted from Proceedings of the Bombay Chamber of Commerce, May 1943).

33 “Memorandum submitted by the All-India Trade Union Congress…”, *Trade Union Record,* IV/5–6 (January–February 1945), p. 39.

34 “Fixing of Labour Conditions by Employers”, *ToI,* 13 April 1946, p. 8.

35 ILOrep 1/1942, pp. 4–6 (special reports on the 3rd Labour Ministers’ Conference and the preceding meeting of the Labour Ministers with employer and worker representatives in January 1942).

36 See also: “Health Insurance Plan for Workers. Provincial Govt.’s Attitude”, *ToI,* 31 October 1944, p. 4.

37 ILOrep 7/1947, pp. 28f (cites proceedings of the Committee of the Bombay Millowners’ Association for January to March 1947).

38 “Health Insurance Scheme. Central Act Likely to be Amended”, *Bombay Chronicle,* 5 December 1950, p. 1; *Report of the Study Group on Social Security,* pp. 24f.

39 *A Question to Trade Unions: On ESI, PF and Pension Schemes*, p. 107.

40 Adarkar quoted a resolution to this effect of a Joint Conference of the (expatriate British) Employers’ Federation of India and the All-India Organisation of Industrial Employers (controlled by Indian big business interests) in September 1940 (Adarkar 1945: 164). See also: ILOrep 8/1940, p. 37 (summarizing a “Letter to the Superintendent of Industries, Delhi, sent by the Secretary, All-India Organisation of Industrial Employers, on 15-8-1940, copy of which was forwarded to this Office”). This line of reasoning was endorsed by the Bombay Millowners’ Association and the Calcutta Chamber of Commerce. Cf. ILOrep 9/1940, p. 26 and ibid., 10/1940, p. 15.

41 “Scheme for Sickness Insurance. ‘Should be on All-India Basis’”, *ToI,* 30 June 1943, p. 6. See also: ILOrep 6/1943, p. 12 (excerpted from Proceedings of the Bombay Chamber of Commerce, May 1943).

42 “Employers’ Federation Pledge Support to Government”, *Bombay Chronicle,* 20 December 1947, p. 3. See also ILOrep 7/1943, p. 12 (cites *Journal of the Indian Merchants’ Chamber*, July 1943).

43 ILOrep 10/1944, pp. 24f (cites “Unofficial Note issued by the Bureau of Public Information, Government of India”, n.d.).

44 “Memorandum submitted by the All-India Trade Union Congress …”, *Trade Union Record,* IV/5–6 (January–February 1945), pp. 38, 40; see also: ILOrep 3/1945, pp. 24f.

45 “Note on the Report prepared by Professor B.P. Adarkar on a Scheme of Health Insurance for Industrial Workers, by Mr. M. Stack and Mr. R. Rao of the International Labour Office”, p. 3, BL: IOR/L/E/8/4948 (“Health Insurance for Industrial Workers, including Sickness Statistics”).

46 *ILG* V/10 (April 1948), pp. 698–701; ILOrep 4/1948, pp. 90–93 (referring to *The Statesman,* 2 and 3 April 1948). For a contemporary analysis of the Act in its final form, see confidential report by K.D. Jones, Labour Adviser to the High Commissioner for the UK in New Delhi, to Secretary, Ministry of Labour and National Service, Overseas Department, London, 18 May 1948, in: BL: IOR/L/E/8/6222.

47 RCLI, *Evidence,* Vol. VII, part 1: Railways, London: H.M.’s Stationary Office, 1931, pp. 53–69; Dilip Subramaniam, “‘No Room for Class Struggle in These National Undertakings’: Providing Social Welfare for Indian State Sector Industrial Workers (circa 1950–2000),” *MAS* 49 (2015), pp. 1526–1579.

48 This issue will be examined more systematically elsewhere. For a perceptive exploration of conflicts over the legal status of “worker” and “employee” in South India see: Karuna (2019).

49 “Speech by the Minister of Labour, Jagjivan Ram, at the inaugural ceremony of the Employees’ State Insurance Corporation”, 6 October 1948, BL: IOR/L/E/8/6222 “The Employees’ State Insurance Act 1948”. See also: “‘India Has Turned the Corner Now’”, *ToI,* 7 October 1948, p. 3.

50 ILOrep 4/1949, pp. 71f (citing *Hindustan Times,* 22 April 1949).

51 Government of India, Films Division, “Dawn of Social Security” (1952).

52 W. Beveridge to D. Monteath, India Office, draft letter, 21 September 1945, BL: IOR/L/E/8/2903 “Social Security in India. Proposed Visit by Sir W. Beveridge”.

53 For a case concerning construction workers see “Road roko: police remove construction workers”, *The Hindu,* 10 February 2011. For a case concerning powerloom workers see “Over 5,000 powerlooms fall silent in Chennimalai”, *The Hindu,* 28 October 2007. For recent cases of contract workers demanding inclusion in the ESI, see, for example, “Workers rampage at DLF site, seek better work conditions”, *The Hindu,* 22 July 2017; “Contract power staff on strike to seek service regularisation”, *The Hindu,* 25 July 2018.

54 See, for instance, “Code on Social Security 2019: Sharp division among trade unions over benefits to workers”, *Business Today,* 13 December 2019, https://www.businesstoday.in/sectors/jobs/code-on-social-security-2019-rss-affiliated-bms-left-affiliated-citu-division-among-trade-unions-over-benefit-to-workers/story/391993.html; Rajat Mishra: “A Quick Look at the Social Security Bill 2019”, *Outlook Money,* 21 December 2019, https://www.outlookindia.com/outlookmoney/talking-money/a-quick-look-at-the-social-security-bill-2019-4069.
